# Association between leisure-time physical activity and incident cancer risk: a nationwide population-based cohort study

**DOI:** 10.1186/s40798-024-00780-y

**Published:** 2024-10-25

**Authors:** Yun-Ju Lai, Chun-Chieh Wang, Yu-Kai Lin, Mei-Ju Chen, Yi-Sheng Chou, Chu-Chieh Chen, Chieh-Yu Liu, Shang-Jung Wu, Li-Fei Hsu, Jia-Hua Li, Yung-Feng Yen

**Affiliations:** 1 School of Medicine, National Yang Ming Chiao Tung University, Taipei, Taiwan; 2 Division of Endocrinology and Metabolism, Department of Internal Medicine, Puli Branch of Taichung Veterans General Hospital, Nantou, Taiwan; 3 Department of Exercise Health Science, National Taiwan University of Sport, Taichung, Taiwan; 4 Department of Health Care Management, National Taipei University of Nursing and Health Sciences, Taipei, Taiwan; 5 Division of Chest Medicine, Department of Internal Medicine, Puli Branch of Taichung, Veterans General Hospital, Nantou, Taiwan; 6 Department of Eldercare, Central Taiwan University of Science and Technology Taichung, Taichung, Taiwan; 7 Department of Health and Welfare, College of City Management, University of Taipei, Taipei City, Taiwan; 8 Family Medicine Department, Heping Fuyou Branch, Taipei City Hospital, Taipei, Taiwan; 9 Department of Hematology and Oncology, Renai Branch, Taipei City Hospital, Taipei, Taiwan; 10 Institute of Emergency and Critical Care Medicine, National Yang Ming Chiao Tung University, Taipei, Taiwan; 11 Department of Nursing, Puli Branch of Taichung Veterans General Hospital, Nantou, Taiwan; 12 Section of Infectious Diseases, Yangming Branch, Taipei City Hospital, Taipei, Taiwan; 13 Institute of Public Health, National Yang Ming Chiao Tung University, Taipei, Taiwan; 14 Department of Education and Research, Taipei City Hospital, Taipei City, Taiwan; 15 Department of Psychology and Counseling, University of Taipei, Taipei, Taiwan

**Keywords:** Physical activity, Cancer, Risk, Prevention, Cohort study, Taiwan

## Abstract

**Background:**

The effects of physical activity on the development of different types of cancers have not been comprehensively studied. This nationwide, population-based cohort study investigated the effects of leisure-time physical activity (LTPA) on the development of different types of cancer in Taiwanese adults. A total of 67,890 adult participants (≥ 18 y old) from five rounds (2001, 2005, 2009, 2013, and 2017) of the Taiwan National Health Interview Survey were included. LTPA was measured as the metabolic equivalent of task (MET) expenditure per week and was classified as inactive (< 1 MET-h), low (1-7.49 MET-h), or high (≥ 7.5 MET-h). The LTPA and other covariates were collected through in-person interviews at baseline. New-onset cancer was ascertained from histopathological reports. The Fine-Gray sub-distribution method, with death as a competing risk, was used to determine the impact of LTPA on incident cancer risk.

**Results:**

During the 844,337 person-years of follow-up, 4,435 individuals developed cancer. Compared to inactive adults, individuals engaging in high levels of LTPA (≥ 7.5 MET-h/week) were significantly associated with a reduced risk of developing cancer (adjusted hazard ratio [aHR] = 0.93; 95% confidence interval [CI] = 0.87–0.99). However, those with low levels of LTPA (1-7.49 MET-h/week) did not exhibit a significant association with a reduced risk of developing cancer (aHR = 1.00; 95% CI = 0.92–1.10). When considering specific types of cancers, participants with high levels of LTPA (≥ 7.5 MET-h/week) had a significantly lower risk of developing bladder cancer (aHR = 0.68; 95% CI = 0.47–0.99), cervical cancer (aHR = 0.48; 95% CI = 0.24–0.95), and thyroid cancer (aHR = 0.64; 95% CI = 0.44–0.93).

**Conclusions:**

Our findings suggest that high LTPA (≥ 7.5 MET-h/week) is significantly associated with a low risk of incident bladder, cervical, and thyroid cancers.

**Supplementary Information:**

The online version contains supplementary material available at 10.1186/s40798-024-00780-y.

## Key points

This prospective cohort study with a mean follow-up of 12.4 years found that high leisure-time physical activity (≥ 7.5 MET-h/week) was significantly associated with a lower risk of incident cancer, including bladder, cervical, and thyroid cancer.

Healthcare professionals should encourage inactive adults to perform leisure-time physical activities to reduce cancer risk.

**Supplementary Information:**

The online version contains supplementary material available at 10.1186/s40798-024-00780-y.

## Background

Cancer is the leading cause of death worldwide [1]. In 2016, approximately 17.2 million new cancer cases were reported globally, corresponding to an incident rate of 229.3 per 100,000 individuals [2]. In Taiwan, 70,534 new cancer cases were recorded in 2016, equating to an incident rate of 299.6 per 100,000 individuals [3]. The World Health Organization estimated that 30–50% of new incident caners could be prevented by addressing risk factors or adopting healthy lifestyles [1].

Physical activity is a modifiable lifestyle factor associated with a lower risk of all-cause, cardiovascular disease (CVD), and cancer-related mortality [4–6]. A prior report showed that leisure-time physical activity (LTPA) ≥ 7.5 metabolic equivalents of task (MET)-h/week, as recommended by the US Physical Activity Guidelines, decreased the risk of all-cause mortality by 27% [4]. Another 2019 meta-analysis revealed that higher pre and postdiagnosis levels of physical activity were associated with improved survival outcomes for patients diagnosed with cancers [6]. Despite these benefits, physical inactivity is highly prevalent globally, with an estimated 31% of worldwide population, 17% population in Southeast Asia, and 53% of the population in the US not attaining recommended levels [7, 8].

Reducing cancer development risk through increased physical activity has received significant attention for many years [9]. A 2019 meta-analysis reported that physical activity was associated with a 10–20% reduction in the relative risk of developing several common cancers, including breast, colon, and bladder cancers [10]. The effect of physical activity on the risk of various types of cancer incidents is mediated by a complex mechanism that involves sex hormones, and a reduction in systemic inflammation, adipokines, insulin and insulin growth factor-1 (IGF-1) [9]. Physical activity in association with weight reduction could decrease the level of estrogen and potentially reduce the risk of breast cancer among postmenopausal women [11]. Regular moderate to vigorous physical activity also could reduce insulin and IGF-1 levels and may decrease the risk of colon cancer [12]. While epidemiological studies have linked physical activity to a reduced risk of breast, colon, and bladder cancers, the evidence for an association between physical activity and haematological, head and neck, cervical, and pancreatic cancers remains limited, mainly because of the lack of studies on these cancers [10].

Understanding the impact of physical activity on the risk of incident cancer will provide important information for future cancer prevention programs. Therefore, we conducted the nationwide population-based cohort study to investigate the effects of physical activity on the risk of incident cancer in Taiwanese adults.

## Methods

### Setting and subjects

The study population derived from five rounds of the National Health Interview Survey (NHIS), a comprehensive national survey conducted in 2001, 2005, 2009, 2013, and 2017 in Taiwan. The NHIS is conducted every four years to aid Taiwan’s public health sector in monitoring the population’s health status [13]. It employs a multi-stage stratified systematic sampling design to select a representative sample of the general Taiwanese population [13]. Townships are stratified by the levels of urbanization and geographic location before sampling, and individuals in sampled villages or lin of townships are selected step by step following the principle of probability proportional to size [13]. A total of 159 interviewers were recruited and trained for this research. After obtaining participants’ informed consent, interviewers conducted in-person interviews to collect participants’ socio-demographic information and health behaviors at the place of their residence. The duration of each interview ranged between 30 min and one hour. The NHIS included three sets of questions designated for three age groups: under 12 years, 12 to 64 years, and 65 years or older. The questionnaire regarding health behaviors for those aged 12 to 64 years and 65 years or older is the same as in the NHIS.

This study included 83,794 participants aged ≥ 18 years from Taiwan’s 2001, 2005, 2009, 2013, and 2017 NHIS (supplementary Fig. 1). Individuals with a history of cancer prior to enrollment (*n* = 1,837) were excluded. Additionally, 233 individuals were interviewed twice, and data were only collected from their initial interview. Furthermore, participants with incomplete covariate information (*n* = 13,834) were excluded. Finally, the study comprised 89,826 individuals. All participants in this cohort study were followed up until a new diagnosis of cancer, mortality, or until December 31, 2020, whichever was earliest. The mortality of study participants was confirmed using the death certificate database of Taiwan [14]. The NHIS dataset was linked to the Registry for Catastrophic Illness of Taiwan to identify participants who received a new cancer diagnosis. In Taiwan, a peer review of pathohistological reports is required before an International Classification of Diseases, 9th Revision, Clinical Modification (ICD-9-CM) or the International Classification of Diseases, 10th Revision, Clinical Modification (ICD-10-CM) code for cancer diagnosis is recorded in the Registry for Catastrophic Illnesses [15].

The Institutional Review Board of the Taichung Veterans General Hospital (No. TVGHIRB-SE22286B) approved the study protocol, and the requirement for informed consent was waived. All related procedures were performed following the relevant national and institutional guidelines as well as those stipulated in the Declaration of Helsinki.

### Outcome measurement

The outcome variable was new-onset cancer, determined from records in the Registry for Catastrophic Illnesses of Taiwan [15]. In Taiwan, individuals diagnosed with cancer, except for those with carcinoma in situ, have the option to apply for a catastrophic illness certificate from the National Health Insurance Administration. Holders of this certificate are relieved from copayment fees for cancer-related healthcare services.

In this study, new-onset cancer was classified into 21 types, confirmed on the basis of pathohistological reports and the corresponding ICD-9 and ICD-10 codes in the Registry for Catastrophic Illness of Taiwan (Supplementary Table 1).

### Exposure variable

The primary exposure variable was LTPA, determined by asking participants the following three close-ended questions: (1) How often do they exercise every week? (2) What type of exercises do they perform? and (3) How long do they exercise for? The questionnaire listed over 30 exercise activities, including walking, swimming, running, cycling, golf, tennis, basketball, dancing, yoga, and hiking. Each activity’s metabolic equivalents (METs) were quantified to measure exercise intensity [16]. We determined the total volume of LTPA in study participants by multiplying the activity intensity (MET) by the duration (hours) per week [16]. The US Physical Activity Guidelines have recommended a minimum of 7.5 MET-h/week of aerobic activity for achieving “substantial” health benefits [4]. Participants in our study were categorized as either inactive (< 1 MET-h/week), low (1–7.49 MET-h/week), or high (≥ 7.5 MET-h/week) based on activity levels.

### Controlling variables

The covariates identified in previous studies as risk factors for cancer [17] were assessed in our analyses, including age, sex, marital status, education level, household income, body mass index (BMI), smoking status, alcohol consumption, intake of vegetables and fruits, and comorbidities. Marital status was categorized as either unmarried, married/cohabiting, or other (e.g., widowed, divorced, separated, or single parent). Educational level was classified as elementary or lower, high school, or university or higher. BMI was categorized as underweight (< 18.5 kg/m^2^), normal (18.5–23.9 kg/m^2^), overweight (24–26.9 kg/m^2^), and obese (≥ 27 kg/m^2^) [18]. Smoking status included never smoking, quit smoking, and currently smoking. Alcohol consumption was classified as never, social (less than once per week), regular (once per week or more, but not to the extent of intoxication), or heavy alcohol use (once per week or more and to the extent of intoxication) [19]. Vegetable and fruit intake was categorized as less than 5 days per week or 5–7 days per week.

This study linked the NHIS database to the National Health Insurance Research Database to identify comorbidities among participants. Chronic comorbidities were confirmed based on ICD-9 and ICD-10 codes, which includes diabetes (ICD-9 code 250; ICD-10 codes E08–E13), chronic kidney disease (CKD; ICD-9 codes 580–587; ICD-10 code N18), chronic obstructive pulmonary disease (COPD; ICD-9 codes 491, 492, and 496; ICD-10 codes J41–J44), and liver cirrhosis (ICD-9 codes 571, 571.2, 571.5, and 571.6; ICD-10 codes K703, K717, and K746). A participant was considered to have a comorbidity only if the condition occurred in an inpatient setting or during three or more outpatient visits [20].

### Statistical analysis

The baseline characteristics of participants with different LTPA levels were compared using one-way analysis of variance (ANOVA) for continuous variables and the Chi-square test for categorical variables. The incidence of cancer per 1000 person-years was calculated for individuals with different LTPA levels.

We used the multivariable Cox proportional hazards model to estimate the association between LTPA and incident cancer risk after adjusting for age, sex, education, household income, BMI, smoking status, alcohol consumption, fruit and vegetable intake, and comorbidities. In these models, the Fine-Gray sub-distribution hazard model, with death from any cause as the competing risk, was used to determine the impact of LTPA on the development of different types of cancer [21]. Adjusted hazard ratios (aHR) with 95% confidence intervals (CI) were reported to show the strength and direction of these associations. All data analyses were performed using SAS software (version 9.4; SAS Institute, Cary, NC, USA).

## Results

### Baseline characteristics

The average age of the 67,780 participants was 43.5 years (range: 18–100 years), and males comprised 49.8% of the subjects.

Table 1 shows the baseline characteristics of the study population according to LTPA level. Approximately 49.7%, 15.4%, and 34.9% of the study participants were classified as inactive, low, or high LTPA, respectively. Compared to inactive adults, those with high LTPA had a lower proportion of obesity (16.7% vs. 18.7%) and a higher proportion of university or higher education (44.2% vs. 26.2%).

**Table 1:** Baseline characteristics of the study population according to the level of leisure time physical activity measured as MET expenditure per week

Characteristics	Total (*n* = 67,890)	Level of leisure time physical activity (MET-h/week) *n* (%)^†^			*P* value
		< 1	1-7.49	≥ 7.5	
		(*n* = 33,757)	(*n* = 10,453)	(*n* = 23,680)	
**Demographics**					
Age in years, mean (SD)	43.5 (16.7)	44.2 (16.8)	41.1 (15.3)	43.7 (17.0)	< 0.001
Sex					
Female	34,053 (50.2)	17,490 (51.8)	5,856 (56.0)	10,707 (45.2)	< 0.001
Male	33,837 (49.8)	16,267 (48.2)	4,597 (44.0)	12,973 (54.8)	
BMI(kg/m^2^)					
Normal weight (18.5–23.9)	35,164 (51.8)	17,146 (50.8)	5,615 (53.7)	12,403 (52.4)	< 0.001
Underweight (< 18.5)	4,375 (6.4)	2,477 (7.3)	720 (6.9)	1,178 (5.0)	
Overweight (24-26.9)	16,498 (24.3)	7,898 (23.4)	2,450 (23.4)	6,150 (26.0)	
Obesity (≥ 27)	11,853 (17.5)	6,236 (18.5)	1,668 (16.0)	3,949 (16.7)	
Marriage status					
Married/cohabiting	40,971 (60.4)	20,657 (61.2)	6,292 (60.2)	14,022 (59.2)	< 0.001
Never married	19,973 (29.4)	9,047 (26.8)	3,300 (31.6)	7,626 (32.2)	
Others^‡^	6,946 (10.2)	4,053 (12.0)	861 (8.2)	2,032 (8.6)	
Education					
≤Elementary school	14,159 (20.9)	8,449 (25.0)	1,592 (15.2)	4,118 (17.4)	< 0.001
High school	29,883 (44.0)	16,470 (48.8)	4,319 (41.3)	9,094 (38.4)	
University or higher	23,848 (35.1)	8,838 (26.2)	4,542 (43.5)	10,468 (44.2)	
Household income					
< US$952/month	20,058 (29.5)	11,964 (35.4)	2,485 (23.8)	5,609 (23.7)	< 0.001
US$952-2,222/month	35,572 (52.4)	17,475 (51.8)	5,656 (54.1)	12,441 (52.5)	
> US$2,222/month	12,260 (18.1)	4,318 (12.8)	2,312 (22.1)	5,630 (23.8)	
Smoking status					
Never	46,760 (68.9)	21,978 (65.1)	7,725 (73.9)	17,057 (72.0)	< 0.001
Current	16,218 (23.9)	9,721 (28.8)	1,996 (19.1)	4,501 (19.0)	
Former	4,912 (7.2)	2,058 (6.1)	732 (7.0)	2,122 (9.0)	
Alcohol					
Non-drinker	39,857 (58.7)	20,412 (60.5)	6,128 (58.6)	13,317 (56.2)	< 0.001
Social	19,068 (28.1)	8,193 (24.3)	3,262 (31.2)	7,613 (32.2)	
Regular	8,671 (12.8)	4,947 (14.7)	1,031 (9.9)	2,693 (11.4)	
Heavy	294 (0.4)	205 (0.6)	32 (0.3)	57 (0.2)	
Vegetables					
<5 days/week	6,072 (8.9)	3,524 (10.4)	902 (8.6)	1,646 (7.0)	< 0.001
5–7 days/week	61,818 (91.1)	30,233 (89.6)	9,551 (91.4)	22,034 (93.1)	
Fruit					
<5 days/week	16,582 (24.4)	9,822 (29.1)	2,405 (23.0)	4,355 (18.4)	< 0.001
5–7 days/week	51,308 (75.6)	23,935 (70.9)	8,048 (77.0)	19,325 (81.6)	
**Comorbidity**					
Diabetes					
No	49,279 (72.6)	24,242 (71.8)	7,865 (75.2)	17,172 (72.5)	< 0.001
Yes	18,611 (27.4)	9,515 (28.2)	2,588 (24.8)	6,508 (27.5)	
Chronic renal failure					
No	61,510 (90.6)	30,443 (90.2)	9,602 (91.9)	21,465 (90.7)	< 0.001
Yes	6,380 (9.4)	3,314 (9.8)	851 (8.1)	2,215 (9.4)	
COPD					
No	52,802 (77.8)	26,099 (77.3)	8,341 (79.8)	18,362 (77.5)	< 0.001
Yes	15,088 (22.2)	7,658 (22.7)	2,112 (20.2)	5,318 (22.5)	
Liver cirrhosis					
No	48,548 (71.5)	24,277 (71.9)	7,608 (72.8)	16,663 (70.4)	< 0.001
Yes	19,342 (28.5)	9,480 (28.1)	2,845 (27.2)	7,017 (29.6)	
Outcome					
New onset of cancer	4,435 (6.5)	2,223 (6.6)	633 (6.1)	1,579 (6.7)	0.093
Follow-up years, mean (SD)	12.4 (5.7)	12.2 (5.9)	12.9 (5.4)	12.6 (5.5)	< 0.001

*MET* metabolic equivalent of task, *SD* standard deviation, *BMI* body mass index, *COPD* chronic obstructive pulmonary disease. ^†^Unless stated otherwise

^‡^Others: widowed, divorced, separated, or single parent

During the 844,337 person-years of follow-up, 4,435 individuals developed new-onset cancer, including 2,223 (6.6%) participants with inactive LTPA, 633 (6.1%) with low LTPA and 1,579 (6.7%) with high LTPA.

### Association between leisure-time physical activity and incident cancer risk

The Fine–Gray sub-distribution hazard model showed that compared to inactive adults, individuals with high levels of LTPA exhibited a significantly reduced risk of incident cancer (aHR = 0.93; 95% CI = 0.87–0.99). However, those with low levels of LTPA did not show a significantly decreased risk of incident cancer (aHR = 1.00; 95% CI = 0.92–1.10) (Table 2). The independent risk factors for incident cancer were older age, current and former smokers, regular and heavy alcohol consumption, and liver cirrhosis. In addition, compared to participants with a household income < US $952/month, those with a household income > US $2,222/month had a lower risk of incident cancer. The interaction between LTPA and other covariates was not significant in the multivariate analysis.

**Table 2:** Univariates and multivariate analyses of risk factors for incident cancer

Factors	*N*	Events, *n*	Person-years	IR^a^	Univariate analysis		Multivariates analysis
					HR (95% CI)		AHR (95% CI)
**Level of LTPA (MET-h/week)**							
Inactive (< 1)	33,757	2223	411933.02	5.40	1		1
Low (1-7.49)	10,453	633	135263.62	4.68	0.87 (0.79–0.95)^**^		1.00 (0.92–1.10)
High (≥ 7.5)	23,680	1579	297139.9	5.31	0.99 (0.92–1.05)		0.93 (0.87–0.99)^*^
Age (per 10-year increase)					1.68 (1.65–1.71)^***^		1.65 (1.60–1.69)^***^
Sex							
Female	34,053	2024	424247.8	4.77	1		1
Male	33,837	2411	420088.75	5.74	1.20 (1.13–1.28)^***^		1.04 (0.96–1.12)
BMI(kg/m^2^)							
Normal weight (18.5–23.9)	35,164	2142	449317.69	4.77	1		1
Underweight (< 18.5)	4375	167	56533.72	2.95	0.62 (0.53–0.72)^***^		0.93 (0.80–1.09)
Overweight (24-26.9)	16,498	1278	200625.32	6.37	1.35 (1.26–1.44)^***^		1.02 (0.95–1.09)
Obesity (≥ 27)	11,853	848	137859.81	6.15	1.31 (1.21–1.42)^***^		1.03 (0.95–1.12)
Marriage status							
Married/cohabiting	40,971	3350	510001.68	6.57	1		1
Never married	19,973	434	263020.22	1.65	0.25 (0.23–0.28)^***^		0.77 (0.68–0.86)^***^
Others^‡^	6946	651	71314.64	9.13	1.43 (1.32–1.56)^***^		0.99 (0.90–1.08)
Education							
≤Elementary school	14,159	1779	161905.41	10.99	1		1
High school	29,883	1781	388735.76	4.58	0.41 (0.38–0.44)^***^		1.01 (0.93–1.09)
University or higher	23,848	875	293695.38	2.98	0.27 (0.25–0.29)^***^		1.02 (0.92–1.12)
Household income							
< US$952/month	20,058	1760	236072.04	7.46	1		1
US$952-2,222/month	35,572	2038	449512.66	4.53	0.60 (0.57–0.64)^***^		0.94 (0.88–1.01)
> US$2,222/month	12,260	637	158751.84	4.01	0.53 (0.49–0.58)^***^		0.86 (0.78–0.95)^**^
Smoking status							
Never	46,760	2742	584886.77	4.69	1		1
Currentr	16,218	1268	208908.86	6.07	1.29 (1.21–1.38)^***^		1.38 (1.27–1.50)^***^
Former	4912	425	50540.92	8.41	1.87 (1.69–2.07)^***^		1.27 (1.13–1.42)^***^
Alcohol							
Non-drinker	39,857	2698	507163.25	5.32	1		1
Social	19,068	881	224326.11	3.93	0.76 (0.70–0.82)^***^		0.97 (0.89–1.05)
Regular	8671	827	109321.86	7.56	1.43 (1.32–1.55)^***^		1.24 (1.14–1.36)^***^
Heavy	294	29	3525.32	8.23	1.56 (1.09–2.26)^*^		1.46 (1.01–2.11)^*^
Vegetables							
<5 days/week	6072	432	96796.75	4.46	1		1
5–7 days/week	61,818	4003	747539.8	5.35	1.25 (1.13–1.38)^***^		0.92 (0.83–1.02)
Fruit							
<5 days/week	16,582	1225	256039.7	4.78	1		1
5–7 days/week	51,308	3210	588296.85	5.46	1.20 (1.12–1.28)^***^		1.03 (0.96–1.11)
**Comorbidity**							
Diabetes	18,611	1842	230032.1	8.01	1.90 (1.79–2.01)^***^		0.97 (0.91–1.04)
Chronic renal failure	6380	752	76048.49	9.89	2.07 (1.92–2.24)^***^		0.94 (0.87–1.02)
COPD	15,088	1523	184076.48	8.27	1.88 (1.77-2.00)^***^		1.01 (0.94–1.08)
Liver cirrhosis	19,342	1874	241547.78	7.76	1.82 (1.72–1.93)^***^		1.41 (1.33–1.50)^***^

^*^<0.05; ^**^<0.01; ^***^<0.001

^a^Cancer incidence per 1000 person-years

^‡^Others: widowed, divorced, separated, or serving as a single parent

*IR* incidence rate, *AHR* adjusted hazard ratio, *CI* confident interval, *LTPA* leisure time physical activity, *MET* metabolic equivalent of task, *COPD* chronic obstructive pulmonary disease.

We conducted a subgroup analysis to evaluate the association between LTPA and cancer risk, stratifying participants by age (≥ 50 years or < 50 years), BMI categories, and liver cirrhosis status. The analysis revealed that high levels of LTPA appeared to correlate with a reduced cancer risk across both age groups and in individuals with and without liver cirrhosis. However, these associations were not statistically significant. Additionally, high levels of LTPA were significantly associated with a decreased risk of developing cancer in individuals with normal weight. This association was not significant for underweight, overweight, or obese individuals (supplementary Table 2).

### Association of leisure-time physical activity with specific cancer type risk

**Fig. 1:**
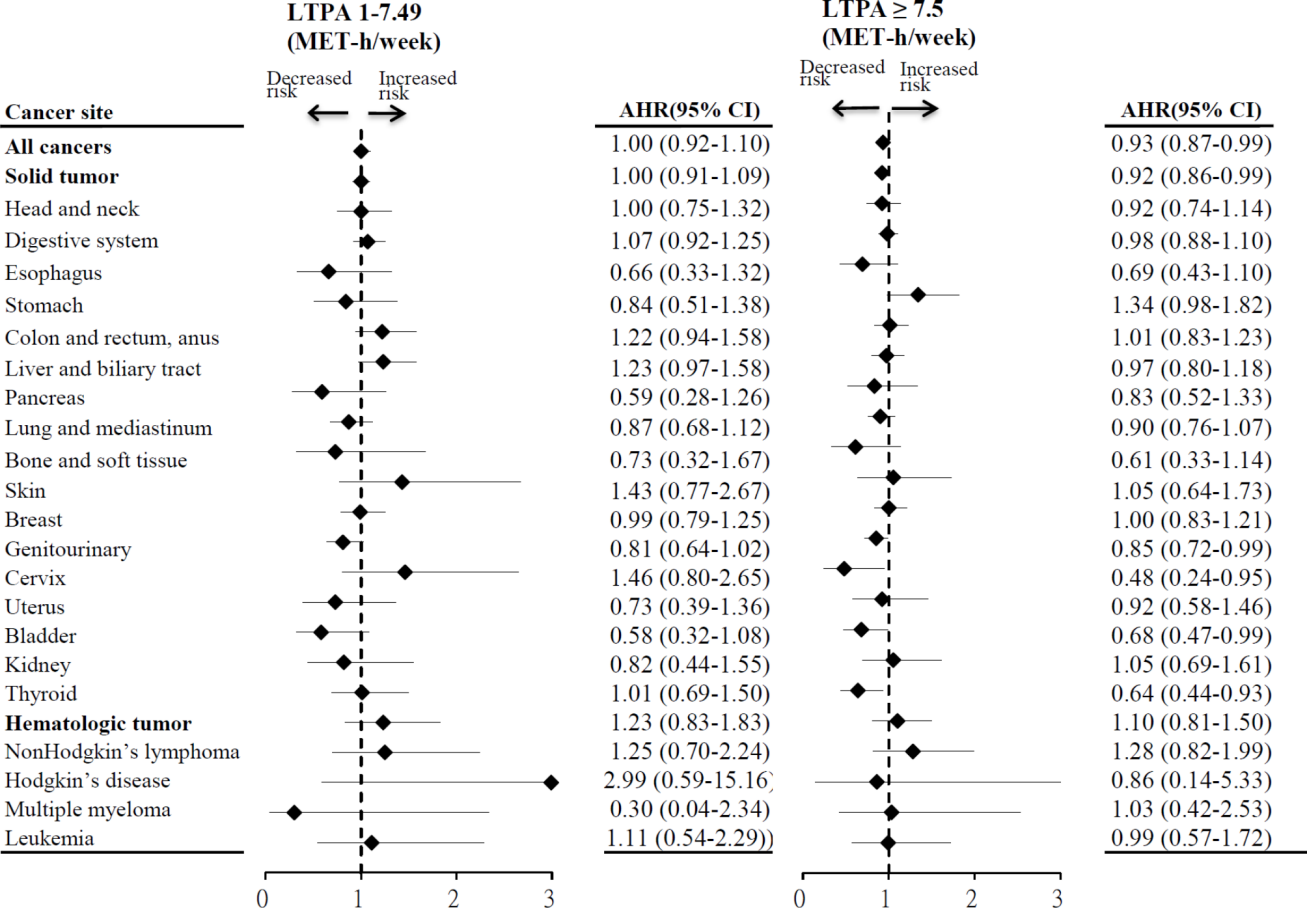
Association of leisure time physical activity with the risk of specific cancer types Abbreviations: *LTPA* leisure-time physical activity, *MET* metabolic equivalents of task, *AHR* adjusted hazard ratio, *CI* confidence interval

Figure 1 shows the association between LTPA and the risk of specific cancer types. Compared to inactive adults, those with high LTPA had a significantly lower risk of incident bladder (aHR = 0.68; 95% CI = 0.47–0.99), cervical (aHR = 0.48; 95% CI = 0.24–0.95), and thyroid cancer (aHR = 0.64; 95% CI = 0.44–0.93). The incidence rates of each specific type of cancer among individuals with varying levels of LTPA are presented in the supplementary Table 3.

We conducted the subgroup analysis to determine the impact of LTPA on the risk of bladder cancer after stratifying the participants based on their smoking status. The subgroup analysis revealed that high levels of LTPA were significantly linked to a decreased risk of developing bladder cancer in non-smokers, current smokers, and former smokers. However, this association was not significant for any of these groups regarding the reduction in bladder cancer incidence (supplementary Table 4).

## Discussion

In this prospective cohort study with a mean follow-up of 12.4 years, we found that individuals engaging in high LTPA (≥ 7.5 MET-h/week) were significantly associated with a lower risk of incident cancer than inactive adults. When considering the effect of physical activity on specific cancer types, participants with ≥ 7.5 MET-hours per week of activity showed a significantly reduced risk of developing bladder, cervical, and thyroid cancers.

Physical activity is a lifestyle factor that can be modified, and its benefits in lowering cancer risk have garnered greater attention [9]. A prior randomized controlled trial with 439 overweight and obese postmenopausal women demonstrated that aerobic exercise combined with weight loss was associated with reduced estrogen levels and potentially decreased breast cancer risk [11]. A Swedish cohort study revealed that individuals engaged in heavy occupational physical activity had a lower risk of bladder cancer than those with sedentary occupational physical activity [22]. Another US study including 128 patients with cervical cancer and 512 controls found that patients with cervical cancer had significantly increased odds of reporting abstinence from recreational physical activity compared to non-cancer controls [23].

Colon cancer is the third most commonly occurring cancer in men and women [24]. The effect of physical activity on the risk of colon cancer risk has been reported in the previous studies [25, 26]. A US cohort study involving 29,133 male smokers aged 50–69 years revealed that compared to sedentary workers, individuals with moderate to heavy levels of occupational activity had a reduced risk of distal colon cancer (relative risk [RR], 0.21; CI, 0.09–0.51) but did not have a lower risk of proximal colon cancer (RR, 0.87; CI, 0.40–1.92) [25]. Moreover, there was no significant association between LTPA and colon cancer (active versus sedentary; RR, 0.82; CI, 0.59–1.13) [25]. Another cohort study conducted in Sweden, which included 45,906 men, found that individuals who engaged in 60 min or more of daily LTPA had a significantly lower risk of developing colon cancer compared to those who engaged in less than 10 min per day (AHR 0.57; 95% CI 0.41–0.79) [26]. In our cohort study of 67,890 individuals with a mean follow-up of 12.4 years, we found that participants engaging in ≥ 7.5 MET-h/week of LTPA had a significantly reduced risk of developing cancers such as bladder, cervical, and thyroid malignancies, compared to inactive adults. However, no significant reduction in risk was observed for colon cancer. The diverse definitions of physical activity and variations in study populations may explain the discrepancies in findings regarding the association between physical activity and colon cancer risk in our study compared to previous reports [25, 26].

This cohort study found that high LTPA decreased the risk of incident cancer by 7%. The physical activity-related reduction of sex hormones, adipokines, and proinflammatory cytokines and improvement of insulin resistance may explain the lower risk of incident cancer in individuals with high levels of physical activity. A previous Women’s Health Initiative Dietary Modification Trial revealed that increased physical activity was associated with a lower amount of free circulating active sex hormone [27], potentially reducing the risk of hormone-dependent cancers. While obesity is a known risk factor for cancer [28], our study did not observe a direct association between obesity and increased cancer risk. However, several prior studies have shown that physical activity can reduce the risk of sarcopenic obesity and body fat, which leads to lower levels of adipokines and pro-inflammatory cytokines, such as TNF-α, IL-1β, and IL-6 [29–31]. These changes may help prevent tumor cell proliferation, survival, and migration [32].

Improvements in insulin resistance due to physical activity may also contribute to the reduced cancer risk. A Canadian randomized controlled trial implementing a 1-year physical activity intervention showed that physical activity improved insulin resistance and reduced hyperinsulinemia in the intervention group [33], which could inhibit angiogenesis, apoptosis, and subsequently decrease the risk of cancer development [34]. Since physical activity is a modifiable lifestyle behavior, the findings in this study suggest that healthcare professionals should encourage inactive adults to perform LTPA to reduce cancer risk.

This study has several strengths. It was conducted on the general population from a nationally representative sample, with greater generalizability. The NHIS was designed and executed by an experienced national survey team with quality control over interviews [35]. Additionally, detailed personal information collected at baseline enabled adjustment for major risk factors of cancer. Finally, as this study included comprehensive information on mortality by linking the NHIS dataset with the death certificate database of Taiwan [14], we used the Fine-Gray sub-distribution method [21] with death as the competing risk to precisely estimate the impact of physical activity on cancer development.

Nonetheless, some limitations should be considered when interpreting the findings of this nationwide population-based study. First, leisure-time physical activity was evaluated at baseline and changes in physical activity over the study period were not assessed. Further studies are required to evaluate the time-varying effects of physical activity on cancer development. Second, cancer is a multifactorial disease, wherein genetics may play dynamic roles in its pathogenesis. However, the data regarding susceptibility genes, such as CYP17 for breast cancer and GSTT1 for colorectal cancer, was not available [36]. Third, the diagnosis of new-onset cancer was determined by the Taiwan Registry for Catastrophic Illness, confirmed through patients’ pathohistological reports. However, the Taiwan Catastrophic Illness dataset did not record the cancer stage. Finally, the external validity of the findings may also be a concern, as almost all participants in this cohort study were Taiwanese. Therefore, the generalizability of the results to non-Asian ethnic groups requires further investigation.

## Conclusions

Our cohort study showed that, compared to inactive adults, individuals with high levels of LTPA (≥ 7.5 MET-h/week) exhibited a significantly reduced risk of incident cancer. However, those with low levels of LTPA (1–7.49 MET-h/week) did not show a significantly decreased risk of incident cancer. When considering specific types of cancers, participants with high levels of LTPA (≥ 7.5 MET-h/week) had a significantly reduced risk of developing bladder cancer, cervical cancer, and thyroid cancer. Given that physical activity is a lifestyle factor that can be changed, the findings of our study indicate that healthcare professionals should promote participation in LTPA among inactive adults to reduce their risk of cancer. Further research is necessary to elucidate the mechanisms underlying the influence of LTPA on the risk of various types of cancers.

## Electronic Supplementary Material

Below is the link to the electronic supplementary material.

Supplementary Material 1

Supplementary Material 2

Supplementary Fig. 1. Flow diagram demonstrating selection of the study population. Abbreviations: *NHIS* National Health Interview Survey

## Data Availability

Data are available upon reasonable request.

## Abbreviations

CVD: Cardiovascular disease
LTPA: Leisure-time physical activity
NHIS: National Health Interview Survey
METs: Metabolic equivalent of tasks
ICD-9-CM: International Classification of Diseases, 9th Revision, Clinical Modification
ICD-10-CM: International Classification of Diseases, 10th Revision, Clinical Modification
HR: Hazard ratios
CI: Confidence intervals
