# Supplementary material for: Association between leisure-time physical activity and incident cancer risk: a nationwide population-based cohort study
Source: Sports Med Open. 2024 Oct 25;10:116. doi: 10.1186/s40798-024-00780-y (PMC11511801; doi:10.1186/s40798-024-00780-y)
Supplement: Supplementary file 2 — Supplementary Material 2 [file 40798_2024_780_MOESM2_ESM.docx]

**Association between Leisure-time Physical Activity and Incident Cancer Risk: A Nationwide Population-based Cohort Study**

**Yun-Ju Lai^1,2,3,4^．Chun-Chieh Wang^5,6^．Yu-Kai Lin^7^．****Mei-Ju Chen^4,8^．Yi-Sheng Chou^1,9,10^．Chu-Chieh Chen^4^．****Chieh-Yu Liu^4^．****Shang-Jung Wu^11^．Li-Fei Hsu^12^．****Jia-Hua Li^4^．Yung-Feng Yen^12,13,14,15*^**

^1^School of Medicine, National Yang Ming Chiao Tung University, Taipei, Taiwan

^2^Division of Endocrinology and Metabolism, Department of Internal Medicine, Puli Branch of Taichung Veterans General Hospital, Nantou, Taiwan

^3^Department of Exercise Health Science, National Taiwan University of Sport, Taichung, Taiwan

^4^Department of Health Care Management, National Taipei University of Nursing and Health Sciences, Taipei, Taiwan

^5^Division of Chest Medicine, Department of Internal Medicine, Puli Branch of Taichung Veterans General Hospital, Nantou, Taiwan

^6^Department of Eldercare, Central Taiwan University of Science and Technology Taichung, Taiwan

^7^Department of Health and Welfare, College of City Management, University of Taipei, Taiwan

^8^Family Medicine Department, Taipei City Hospital, Heping Fuyou Branch, Taipei, Taiwan

^9^Department of Hematology and Oncology, Taipei City Hospital, Renai Branch, Taipei, Taiwan

^10^Institute of Emergency and Critical Care Medicine, National Yang Ming Chiao Tung University, Taipei, Taiwan

^11^Department of Nursing, Puli Branch of Taichung Veterans General Hospital, Nantou, Taiwan

^12^Section of Infectious Diseases, Taipei City Hospital, Yangming Branch, Taipei, Taiwan

^13^Institute of Public Health, National Yang Ming Chiao Tung University, Taipei, Taiwan

^14^Department of Education and Research, Taipei City Hospital, Taiwan

^15^Department of Psychology and Counseling, University of Taipei, Taipei, Taiwan

Correspondence: Yung-Feng Yen

Section of Infectious Diseases, Taipei City Hospital, Taipei City Government, Taipei, Taiwan, No.145, Zhengzhou Rd., Datong Dist., Taipei City 10341, Taiwan (Tel: + 886-2-2835-3456; e-mail: [yfyen1@gmail.com](mailto:yfyen1@gmail.com))

**Funding**

This study was supported by the Department of Health, Taipei City Government (No. 11101-62-042 and 11201-62-023). The funders had no role in the design and conduct of the study; collection, management, analysis, and interpretation of the data; preparation, review, or approval of the manuscript; and decision to submit the manuscript for publication.

**Competing interests**

The authors declare no conflict of interest.

| **Supplementary table 1**  International classification of diseases, ninth and tenth revision, clinical modification codes for cancer | | |
| --- | --- | --- |
| **Cancer site** | **ICD-9-CM code** | **ICD-10-CM code** |
| **All cancers** | 140~208 | C, D03 |
| **Solid tumor** | 140~199 | C00-C80, D0 |
| Head and neck | 140-149,160-161 | C00-C14,C30-C32 |
| Digestive system | 150-159 | C15-C26 |
| Esophagus | 150 | C15 |
| Stomach | 151 | C16 |
| Colon and rectum, anus | 153–154 | C18, C21 |
| Liver and biliary tract | 155-156 | C22, C23, C24 |
| Pancreas | 157 | C25 |
| Lung and mediastinum | 162-165 | C33,C34,C37,C38,C39 |
| Bone and soft tissue | 170-171,158 | C40,C41, C47-C49 |
| Skin | 172-173 | D04, C43-C44 |
| Breast | 174, 175 | C50,C55 |
| Genitourinary | 180-189 | C51-C68 |
| Cervix | 180 | C53 |
| Uterus | 182,179 | C54 |
| Bladder | 188 | C67 |
| Kidney | 189 | C64-C65 |
| Thyroid | 193 | C73 |
| **Hematologic malignancies** | 200-208 | C81-C96 |
| NonHodgkin’s lymphoma | 200,202 | C82,C83,C85 |
| Hodgkin’s disease | 201 | C81 |
| Multiple myeloma | 203 | C90 |
| Leukemia | 204-208 | C91-C95 |
| ICD-9-CM = International classification of diseases, ninth revision, clinical modification; ICD-10-CM = International classification of diseases, tenth revision, clinical modification. | | |

| **Supplementary table 2**  Subgroup analysis for the association of leisure time physical activity with the risk of cancer | | |
| --- | --- | --- |
| Study subgroups | LTPA 1-7.49 (MET-h/week) | LTPA ≥ 7.5 (MET-h/week) |
|  | AHR (95% CI) | AHR (95% CI) |
| Aged 18-49 (n=44,790) | 1.01 (0.88-1.16) | 0.96 (0.86-1.08) |
| Aged ≥50 (n=8785) | 1.00 (0.89-1.12) | 0.99 (0.91-1.07) |
| Underweight individuals (n=4,375) | 0.87 (0.52-1.43) | 1.04 (0.73-1.49) |
| Normal weight individuals (n=35,164) | 0.97 (0.85-1.10) | 0.90 (0.82-0.99)^*^ |
| Overweight individuals (n=16,498) | 1.05 (0.89-1.24) | 0.96 (0.85-1.09) |
| Obese individuals (n=11,853) | 1.06 (0.87-1.29) | 0.93 (0.80-1.09) |
| Patients with liver cirrhosis (n=19,342) | 0.96 (0.83-1.10) | 0.92 (0.83-1.02) |
| Patients without liver cirrhosis (n=48,548) | 1.04 (0.93-1.17) | 0.94 (0.86-1.03) |
| ^*^<.05 | | |
| Abbreviation: LTPA, leisure time physical activity; AHR, adjusted hazard ratio; CI, confident interval. | | |

| **Table 3A**  Multivariate analyses of risk factors for incident solid tumor | | | |
| --- | --- | --- | --- |
| Factors | Solid tumor | IR^a^ | Multivariates analysis^b^ |
|  | Events, n |  | AHR (95% CI) |
| **Level of LTPA (MET-h/week)** |  |  |  |
| Inactive (<1) | 2136 | 5.19 | 1 |
| Low (1-7.49) | 603 | 4.46 | 1.00 (0.91-1.09) |
| High (≥ 7.5) | 1501 | 5.05 | 0.92 (0.86-0.99)^*^ |
| ^*^<.05 | | | |
| ^a^Cancer incidence per 1000 person-years. | | | |
| ^b^Adjusting for age, sex, education, household income, BMI, smoking status, alcohol consumption, fruit and vegetable intake, and comorbidities. | | | |
| IR, incidence rate; AHR, adjusted hazard ratio; CI, confident interval; LTPA, leisure time physical activity; MET, metabolic equivalent of task. | | | |

| **Supplementary table 3B**  Multivariate analyses of risk factors for incident head and neck malignancy | | | | |
| --- | --- | --- | --- | --- |
| Factors | Head and neck malignancy | IR^a^ | Multivariates analysis^b^ |  |
|  | Events, n |  | AHR (95% CI) |  |
| **Level of LTPA (MET-h/week)** |  |  |  |  |
| Inactive (<1) | 270 | 0.66 | 1 |  |
| Low (1-7.49) | 59 | 0.44 | 1.00 (0.75-1.32) |  |
| High (≥ 7.5) | 138 | 0.46 | 0.92 (0.74-1.14) |  |
| ^*^<.05 | | | |  |
| ^a^Cancer incidence per 1000 person-years. | | | |  |
| ^b^Adjusting for age, sex, education, household income, BMI, smoking status, alcohol consumption, fruit and vegetable intake, and comorbidities. | | | |  |
| IR, incidence rate; AHR, adjusted hazard ratio; CI, confident interval; LTPA, leisure time physical activity; MET, metabolic equivalent of task. | | | |  |

| **Supplementary table 3C**  Multivariate analyses of risk factors for incident digestive system malignancy | | | |
| --- | --- | --- | --- |
| Factors | Digestive system malignancy | IR^a^ | Multivariates analysis^b^ |
|  | Events, n |  | AHR (95% CI) |
| **Level of LTPA (MET-h/week)** |  |  |  |
| Inactive (<1) | 777 | 1.89 | 1 |
| Low (1-7.49) | 216 | 1.60 | 1.07 (0.92-1.25) |
| High (≥ 7.5) | 571 | 1.92 | 0.98 (0.88-1.10) |
| ^*^<.05 | | | |
| ^a^Cancer incidence per 1000 person-years. | | | |
| ^b^Adjusting for age, sex, education, household income, BMI, smoking status, alcohol consumption, fruit and vegetable intake, and comorbidities. | | | |
| IR, incidence rate; AHR, adjusted hazard ratio; CI, confident interval; LTPA, leisure time physical activity; MET, metabolic equivalent of task. | | | |

| **Supplementary table 3D**  Multivariate analyses of risk factors for incident esophagus tumor | | | |
| --- | --- | --- | --- |
| Factors | Esophagus tumor | IR^a^ | Multivariates analysis^b^ |
|  | Events, n |  | AHR (95% CI) |
| **Level of LTPA (MET-h/week)** |  |  |  |
| Inactive (<1) | 76 | 0.18 | 1 |
| Low (1-7.49) | 9 | 0.07 | 0.66 (0.33-1.32) |
| High (≥ 7.5) | 25 | 0.08 | 0.69 (0.43-1.10) |
| ^*^<.05 | | | |
| ^a^Cancer incidence per 1000 person-years. | | | |
| ^b^Adjusting for age, sex, education, household income, BMI, smoking status, alcohol consumption, fruit and vegetable intake, and comorbidities. | | | |
| IR, incidence rate; AHR, adjusted hazard ratio; CI, confident interval; LTPA, leisure time physical activity; MET, metabolic equivalent of task. | | | |

| **Supplementary table 3E**  Multivariate analyses of risk factors for incident stomach cancer | | | |
| --- | --- | --- | --- |
| Factors | Stomach cancer | IR^a^ | Multivariates analysis^b^ |
|  | Events, n |  | AHR (95% CI) |
| **Level of LTPA (MET-h/week)** |  |  |  |
| Inactive (<1) | 87 | 0.21 | 1 |
| Low (1-7.49) | 19 | 0.14 | 0.84 (0.51-1.38) |
| High (≥ 7.5) | 88 | 0.30 | 1.34 (0.98-1.82) |
| ^*^<.05 | | | |
| ^a^Cancer incidence per 1000 person-years. | | | |
| ^b^Adjusting for age, sex, education, household income, BMI, smoking status, alcohol consumption, fruit and vegetable intake, and comorbidities. | | | |
| IR, incidence rate; AHR, adjusted hazard ratio; CI, confident interval; LTPA, leisure time physical activity; MET, metabolic equivalent of task. | | | |

| **Supplementary table 3F**  Multivariate analyses of risk factors for incident colon and rectum tumor | | | |
| --- | --- | --- | --- |
| Factors | Colon and rectum tumor | IR^a^ | Multivariates analysis^b^ |
|  | Events, n |  | AHR (95% CI) |
| **Level of LTPA (MET-h/week)** |  |  |  |
| Inactive (<1) | 239 | 0.58 | 1 |
| Low (1-7.49) | 79 | 0.58 | 1.22 (0.94-1.58) |
| High (≥ 7.5) | 193 | 0.65 | 1.01 (0.83-1.23) |
| ^*^<.05 | | | |
| ^a^Cancer incidence per 1000 person-years. | | | |
| ^b^Adjusting for age, sex, education, household income, BMI, smoking status, alcohol consumption, fruit and vegetable intake, and comorbidities. | | | |
| IR, incidence rate; AHR, adjusted hazard ratio; CI, confident interval; LTPA, leisure time physical activity; MET, metabolic equivalent of task. | | | |

| **Supplementary table 3F**  Multivariate analyses of risk factors for incident liver and biliary tract malignancy | | | |
| --- | --- | --- | --- |
| Factors | Liver and biliary tract malignancy | IR^a^ | Multivariates analysis^b^ |
|  | Events, n |  | AHR (95% CI) |
| **Level of LTPA (MET-h/week)** |  |  |  |
| Inactive (<1) | 277 | 0.67 | 1 |
| Low (1-7.49) | 86 | 0.64 | 1.23 (0.97-1.58) |
| High (≥ 7.5) | 195 | 0.66 | 0.97 (0.80-1.18) |
| ^*^<.05 | | | |
| ^a^Cancer incidence per 1000 person-years. | | | |
| ^b^Adjusting for age, sex, education, household income, BMI, smoking status, alcohol consumption, fruit and vegetable intake, and comorbidities. | | | |
| IR, incidence rate; AHR, adjusted hazard ratio; CI, confident interval; LTPA, leisure time physical activity; MET, metabolic equivalent of task. | | | |

| **Supplementary table 3G**  Multivariate analyses of risk factors for incident pancreas tumor | | | |
| --- | --- | --- | --- |
| Factors | Pancreas tumor | IR^a^ | Multivariates analysis^b^ |
|  | Events, n |  | AHR (95% CI) |
| **Level of LTPA (MET-h/week)** |  |  |  |
| Inactive (<1) | 49 | 0.12 | 1 |
| Low (1-7.49) | 8 | 0.06 | 0.59 (0.28-1.26) |
| High (≥ 7.5) | 33 | 0.11 | 0.83 (0.52-1.33) |
| ^*^<.05 | | | |
| ^a^Cancer incidence per 1000 person-years. | | | |
| ^b^Adjusting for age, sex, education, household income, BMI, smoking status, alcohol consumption, fruit and vegetable intake, and comorbidities. | | | |
| IR, incidence rate; AHR, adjusted hazard ratio; CI, confident interval; LTPA, leisure time physical activity; MET, metabolic equivalent of task. | | | |

| **Supplementary table 3H**  Multivariate analyses of risk factors for incident lung and mediastinum malignancy | | | |
| --- | --- | --- | --- |
| Factors | Lung and mediastinum malignancy | IR^a^ | Multivariates analysis^b^ |
|  | Events, n |  | AHR (95% CI) |
| **Level of LTPA (MET-h/week)** |  |  |  |
| Inactive (<1) | 338 | 0.82 | 1 |
| Low (1-7.49) | 81 | 0.60 | 0.87 (0.68-1.12) |
| High (≥ 7.5) | 235 | 0.79 | 0.90 (0.76-1.07) |
| ^*^<.05 | | | |
| ^a^Cancer incidence per 1000 person-years. | | | |
| ^b^Adjusting for age, sex, education, household income, BMI, smoking status, alcohol consumption, fruit and vegetable intake, and comorbidities. | | | |
| IR, incidence rate; AHR, adjusted hazard ratio; CI, confident interval; LTPA, leisure time physical activity; MET, metabolic equivalent of task. | | | |

| **Supplementary table 3I**  Multivariate analyses of risk factors for incident bone and soft tissue malignancy | | | |
| --- | --- | --- | --- |
| Factors | Bone and soft tissue malignancy | IR^a^ | Multivariates analysis^b^ |
|  | Events, n |  | AHR (95% CI) |
| **Level of LTPA (MET-h/week)** |  |  |  |
| Inactive (<1) | 32 | 0.08 | 1 |
| Low (1-7.49) | 7 | 0.05 | 0.73 (0.32-1.67) |
| High (≥ 7.5) | 16 | 0.05 | 0.61 (0.33-1.14) |
| ^*^<.05 | | | |
| ^a^Cancer incidence per 1000 person-years. | | | |
| ^b^Adjusting for age, sex, education, household income, BMI, smoking status, alcohol consumption, fruit and vegetable intake, and comorbidities. | | | |
| IR, incidence rate; AHR, adjusted hazard ratio; CI, confident interval; LTPA, leisure time physical activity; MET, metabolic equivalent of task. | | | |

| **Supplementary table 3J**  Multivariate analyses of risk factors for incident skin cancer | | | |
| --- | --- | --- | --- |
| Factors | Skin cancer | IR^a^ | Multivariates analysis^b^ |
|  | Events, n |  | AHR (95% CI) |
| **Level of LTPA (MET-h/week)** |  |  |  |
| Inactive (<1) | 37 | 0.09 | 1 |
| Low (1-7.49) | 14 | 0.10 | 1.43 (0.77-2.67) |
| High (≥ 7.5) | 30 | 0.10 | 1.05 (0.64-1.73) |
| ^*^<.05 | | | |
| ^a^Cancer incidence per 1000 person-years. | | | |
| ^b^Adjusting for age, sex, education, household income, BMI, smoking status, alcohol consumption, fruit and vegetable intake, and comorbidities. | | | |
| IR, incidence rate; AHR, adjusted hazard ratio; CI, confident interval; LTPA, leisure time physical activity; MET, metabolic equivalent of task. | | | |

| **Supplementary table 3K**  Multivariate analyses of risk factors for incident breast cancer | | | |
| --- | --- | --- | --- |
| Factors | Breast cancer | IR^a^ | Multivariates analysis^b^ |
|  | Events, n |  | AHR (95% CI) |
| **Level of LTPA (MET-h/week)** |  |  |  |
| Inactive (<1) | 272 | 0.66 | 1 |
| Low (1-7.49) | 100 | 0.74 | 0.99 (0.79-1.25) |
| High (≥ 7.5) | 203 | 0.68 | 1.00 (0.83-1.21) |
| ^*^<.05 | | | |
| ^a^Cancer incidence per 1000 person-years. | | | |
| ^b^Adjusting for age, sex, education, household income, BMI, smoking status, alcohol consumption, fruit and vegetable intake, and comorbidities. | | | |
| IR, incidence rate; AHR, adjusted hazard ratio; CI, confident interval; LTPA, leisure time physical activity; MET, metabolic equivalent of task. | | | |

| **Supplementary table 3L**  Multivariate analyses of risk factors for incident genitourinary cancer | | | |
| --- | --- | --- | --- |
| Factors | Genitourinary cancer | IR^a^ | Multivariates analysis^b^ |
|  | Events, n |  | AHR (95% CI) |
| **Level of LTPA (MET-h/week)** |  |  |  |
| Inactive (<1) | 375 | 0.91 | 1 |
| Low (1-7.49) | 90 | 0.67 | 0.81 (0.64-1.02) |
| High (≥ 7.5) | 277 | 0.93 | 0.85 (0.72-0.99)^*^ |
| ^*^<.05 | | | |
| ^a^Cancer incidence per 1000 person-years. | | | |
| ^b^Adjusting for age, sex, education, household income, BMI, smoking status, alcohol consumption, fruit and vegetable intake, and comorbidities. | | | |
| IR, incidence rate; AHR, adjusted hazard ratio; CI, confident interval; LTPA, leisure time physical activity; MET, metabolic equivalent of task. | | | |

| **Supplementary table 3M**  Multivariate analyses of risk factors for incident cervical cancer | | | |
| --- | --- | --- | --- |
| Factors | Cervical cancer | IR^a^ | Multivariates analysis^b^ |
|  | Events, n |  | AHR (95% CI) |
| **Level of LTPA (MET-h/week)** |  |  |  |
| Inactive (<1) | 37 | 0.09 | 1 |
| Low (1-7.49) | 16 | 0.12 | 1.46 (0.80-2.65) |
| High (≥ 7.5) | 11 | 0.04 | 0.48 (0.24-0.95)^*^ |
| ^*^<.05 | | | |
| ^a^Cancer incidence per 1000 person-years. | | | |
| ^b^Adjusting for age, sex, education, household income, BMI, smoking status, alcohol consumption, fruit and vegetable intake, and comorbidities. | | | |
| IR, incidence rate; AHR, adjusted hazard ratio; CI, confident interval; LTPA, leisure time physical activity; MET, metabolic equivalent of task. | | | |

| **Supplementary table 3N**  Multivariate analyses of risk factors for incident uterine cancer | | | |
| --- | --- | --- | --- |
| Factors | Uterine cancer | IR^a^ | Multivariates analysis^b^ |
|  | Events, n |  | AHR (95% CI) |
| **Level of LTPA (MET-h/week)** |  |  |  |
| Inactive (<1) | 48 | 0.12 | 1 |
| Low (1-7.49) | 13 | 0.10 | 0.73 (0.39-1.36) |
| High (≥ 7.5) | 33 | 0.11 | 0.92 (0.58-1.46) |
| ^*^<.05 | | | |
| ^a^Cancer incidence per 1000 person-years. | | | |
| ^b^Adjusting for age, sex, education, household income, BMI, smoking status, alcohol consumption, fruit and vegetable intake, and comorbidities. | | | |
| IR, incidence rate; AHR, adjusted hazard ratio; CI, confident interval; LTPA, leisure time physical activity; MET, metabolic equivalent of task. | | | |

| **Supplementary table 3O**  Multivariate analyses of risk factors for incident bladder cancer | | | |
| --- | --- | --- | --- |
| Factors | Bladder cancer | IR^a^ | Multivariates analysis^b^ |
|  | Events, n |  | AHR (95% CI) |
| **Level of LTPA (MET-h/week)** |  |  |  |
| Inactive (<1) | 78 | 0.19 | 1 |
| Low (1-7.49) | 12 | 0.09 | 0.58 (0.32-1.08) |
| High (≥ 7.5) | 45 | 0.15 | 0.68 (0.47-0.99)^*^ |
| ^*^<.05 | | | |
| ^a^Cancer incidence per 1000 person-years. | | | |
| ^b^Adjusting for age, sex, education, household income, BMI, smoking status, alcohol consumption, fruit and vegetable intake, and comorbidities. | | | |
| IR, incidence rate; AHR, adjusted hazard ratio; CI, confident interval; LTPA, leisure time physical activity; MET, metabolic equivalent of task. | | | |

| **Supplementary table 3P**  Multivariate analyses of risk factors for incident kidney cancer | | | |
| --- | --- | --- | --- |
| Factors | Kidney cancer | IR^a^ | Multivariates analysis^b^ |
|  | Events, n |  | AHR (95% CI) |
| **Level of LTPA (MET-h/week)** |  |  |  |
| Inactive (<1) | 51 | 0.12 | 1 |
| Low (1-7.49) | 12 | 0.09 | 0.82 (0.44-1.55) |
| High (≥ 7.5) | 43 | 0.14 | 1.05 (0.69-1.61) |
| ^*^<.05 | | | |
| ^a^Cancer incidence per 1000 person-years. | | | |
| ^b^Adjusting for age, sex, education, household income, BMI, smoking status, alcohol consumption, fruit and vegetable intake, and comorbidities. | | | |
| IR, incidence rate; AHR, adjusted hazard ratio; CI, confident interval; LTPA, leisure time physical activity; MET, metabolic equivalent of task. | | | |

| **Supplementary table 3Q**  Multivariate analyses of risk factors for incident thyroid cancer | | | |
| --- | --- | --- | --- |
| Factors | Thyroid cancer | IR^a^ | Multivariates analysis^b^ |
|  | Events, n |  | AHR (95% CI) |
| **Level of LTPA (MET-h/week)** |  |  |  |
| Inactive (<1) | 90 | 0.22 | 1 |
| Low (1-7.49) | 37 | 0.27 | 1.01 (0.69-1.50) |
| High (≥ 7.5) | 45 | 0.15 | 0.64 (0.44-0.93)^*^ |
| ^*^<.05 | | | |
| ^a^Cancer incidence per 1000 person-years. | | | |
| ^b^Adjusting for age, sex, education, household income, BMI, smoking status, alcohol consumption, fruit and vegetable intake, and comorbidities. | | | |
| IR, incidence rate; AHR, adjusted hazard ratio; CI, confident interval; LTPA, leisure time physical activity; MET, metabolic equivalent of task. | | | |

| **Supplementary table 3R**  Multivariate analyses of risk factors for incident hematologic malignancy | | | |
| --- | --- | --- | --- |
| Factors | Hematologic malignancy | IR^a^ | Multivariates analysis^b^ |
|  | Events, n |  | AHR (95% CI) |
| **Level of LTPA (MET-h/week)** |  |  |  |
| Inactive (<1) | 92 | 0.22 | 1 |
| Low (1-7.49) | 35 | 0.26 | 1.23 (0.83-1.83) |
| High (≥ 7.5) | 85 | 0.29 | 1.10 (0.81-1.50) |
| ^*^<.05 | | | |
| ^a^Cancer incidence per 1000 person-years. | | | |
| ^b^Adjusting for age, sex, education, household income, BMI, smoking status, alcohol consumption, fruit and vegetable intake, and comorbidities. | | | |
| IR, incidence rate; AHR, adjusted hazard ratio; CI, confident interval; LTPA, leisure time physical activity; MET, metabolic equivalent of task. | | | |

| **Supplementary table 3S**  Multivariate analyses of risk factors for incident nonHodgkin’s lymphoma | | | |
| --- | --- | --- | --- |
| Factors | NonHodgkin’s lymphoma | IR^a^ | Multivariates analysis^b^ |
|  | Events, n |  | AHR (95% CI) |
| **Level of LTPA (MET-h/week)** |  |  |  |
| Inactive (<1) | 42 | 0.10 | 1 |
| Low (1-7.49) | 16 | 0.12 | 1.25 (0.70-2.24) |
| High (≥ 7.5) | 44 | 0.15 | 1.28 (0.82-1.99) |
| ^*^<.05 | | | |
| ^a^Cancer incidence per 1000 person-years. | | | |
| ^b^Adjusting for age, sex, education, household income, BMI, smoking status, alcohol consumption, fruit and vegetable intake, and comorbidities. | | | |
| IR, incidence rate; AHR, adjusted hazard ratio; CI, confident interval; LTPA, leisure time physical activity; MET, metabolic equivalent of task. | | | |

| **Supplementary table 3T**  Multivariate analyses of risk factors for incident Hodgkin’s disease | | | |
| --- | --- | --- | --- |
| Factors | Hodgkin’s disease | IR^a^ | Multivariates analysis^b^ |
|  | Events, n |  | AHR (95% CI) |
| **Level of LTPA (MET-h/week)** |  |  |  |
| Inactive (<1) | 3 | 0.01 | 1 |
| Low (1-7.49) | 3 | 0.02 | 2.99 (0.59-15.16) |
| High (≥ 7.5) | 2 | 0.01 | 0.86 (0.14-5.33) |
| ^*^<.05 | | | |
| ^a^Cancer incidence per 1000 person-years. | | | |
| ^b^Adjusting for age, sex, education, household income, BMI, smoking status, alcohol consumption, fruit and vegetable intake, and comorbidities. | | | |
| IR, incidence rate; AHR, adjusted hazard ratio; CI, confident interval; LTPA, leisure time physical activity; MET, metabolic equivalent of task. | | | |

| **Supplementary table 3U**  Multivariate analyses of risk factors for incident multiple myeloma | | | |
| --- | --- | --- | --- |
| Factors | Multiple myeloma | IR^a^ | Multivariates analysis^b^ |
|  | Events, n |  | AHR (95% CI) |
| **Level of LTPA (MET-h/week)** |  |  |  |
| Inactive (<1) | 11 | 0.03 | 1 |
| Low (1-7.49) | 1 | 0.01 | 0.30 (0.04-2.34) |
| High (≥ 7.5) | 11 | 0.04 | 1.03 (0.42-2.53) |
| ^*^<.05 | | | |
| ^a^Cancer incidence per 1000 person-years. | | | |
| ^b^Adjusting for age, sex, education, household income, BMI, smoking status, alcohol consumption, fruit and vegetable intake, and comorbidities. | | | |
| IR, incidence rate; AHR, adjusted hazard ratio; CI, confident interval; LTPA, leisure time physical activity; MET, metabolic equivalent of task. | | | |

| **Supplementary table 3V**  Multivariate analyses of risk factors for incident leukemia | | | |
| --- | --- | --- | --- |
| Factors | Leukemia | IR^a^ | Multivariates analysis^b^ |
|  | Events, n |  | AHR (95% CI) |
| **Level of LTPA (MET-h/week)** |  |  |  |
| Inactive (<1) | 32 | 0.08 | 1 |
| Low (1-7.49) | 10 | 0.07 | 1.11 (0.54-2.29)) |
| High (≥ 7.5) | 24 | 0.08 | 0.99 (0.57-1.72) |
| ^*^<.05 | | | |
| ^a^Cancer incidence per 1000 person-years. | | | |
| ^b^Adjusting for age, sex, education, household income, BMI, smoking status, alcohol consumption, fruit and vegetable intake, and comorbidities. | | | |
| IR, incidence rate; AHR, adjusted hazard ratio; CI, confident interval; LTPA, leisure time physical activity; MET, metabolic equivalent of task. | | | |

| **Supplementary table 4**  Subgroup analysis for the association of leisure time physical activity with the risk of cancer after stratifying the participants based on their smoking categories. | | | |
| --- | --- | --- | --- |
| Study subgroups | LTPA 1-7.49 (MET-h/week) | LTPA ≥ 7.5 (MET-h/week) | |
|  | AHR (95% CI) | AHR (95% CI) | |
| Never smoker (n=46,760) | 0.43 (0.18-1.00) | 0.65 (0.40-1.04) | |
| Current smoker (n=16,218) | 0.63 (0.19-2.12) | 0.81 (0.39-1.70) | |
| Former smoker (n=4,912) | 1.39 (0.35-5.55) | 0.48 (0.13-1.72) | |
| Abbreviation: LTPA, leisure time physical activity; AHR, adjusted hazard ratio; CI, confident interval. | | |  |
