# Supplementary material for: Association between leisure-time physical activity and incident cancer risk: a nationwide population-based cohort study
Source: Sports Med Open. 2024 Oct 25;10:116. doi: 10.1186/s40798-024-00780-y (PMC11511801; doi:10.1186/s40798-024-00780-y)
Supplement: Supplementary file 3 — Supplementary Fig. 1. Flow diagram demonstrating selection of the study population. Abbreviations: NHIS National Health Interview Survey [file 40798_2024_780_MOESM3_ESM.pdf]

[9]Department of Hematology and Oncology, Taipei City Hospital, Renai Branch, Taipei, Taiwan

[10]Institute of Emergency and Critical Care Medicine, National Yang Ming Chiao Tung University, Taipei, Taiwan

[11]Department of Nursing, Puli Branch of Taichung Veterans General Hospital, Nantou, Taiwan

[12]Section of Infectious Diseases, Taipei City Hospital, Yangming Branch, Taipei, Taiwan

[13]Institute of Public Health, National Yang Ming Chiao Tung University, Taipei, Taiwan

[14]Department of Education and Research, Taipei City Hospital, Taiwan

[15]Department of Psychology and Counseling, University of Taipei, Taipei, Taiwan

Correspondence: Yung-Feng Yen

Section of Infectious Diseases, Taipei City Hospital, Taipei City Government, Taipei, Taiwan, No.145, Zhengzhou Rd., Datong Dist., Taipei City 10341, Taiwan (Tel: + 886-2-2835-3456; e-mail: yfyen1@gmail.com)

Funding

This study was supported by the Department of Health, Taipei City Government (No. 11101-62-042 and 11201-62-023). The funders had no role in the design and conduct of the study; collection, management, analysis, and interpretation of the data;

preparation, review, or approval of the manuscript; and decision to submit the manuscript for publication.

Competing interests

The authors declare no conflict of interest.

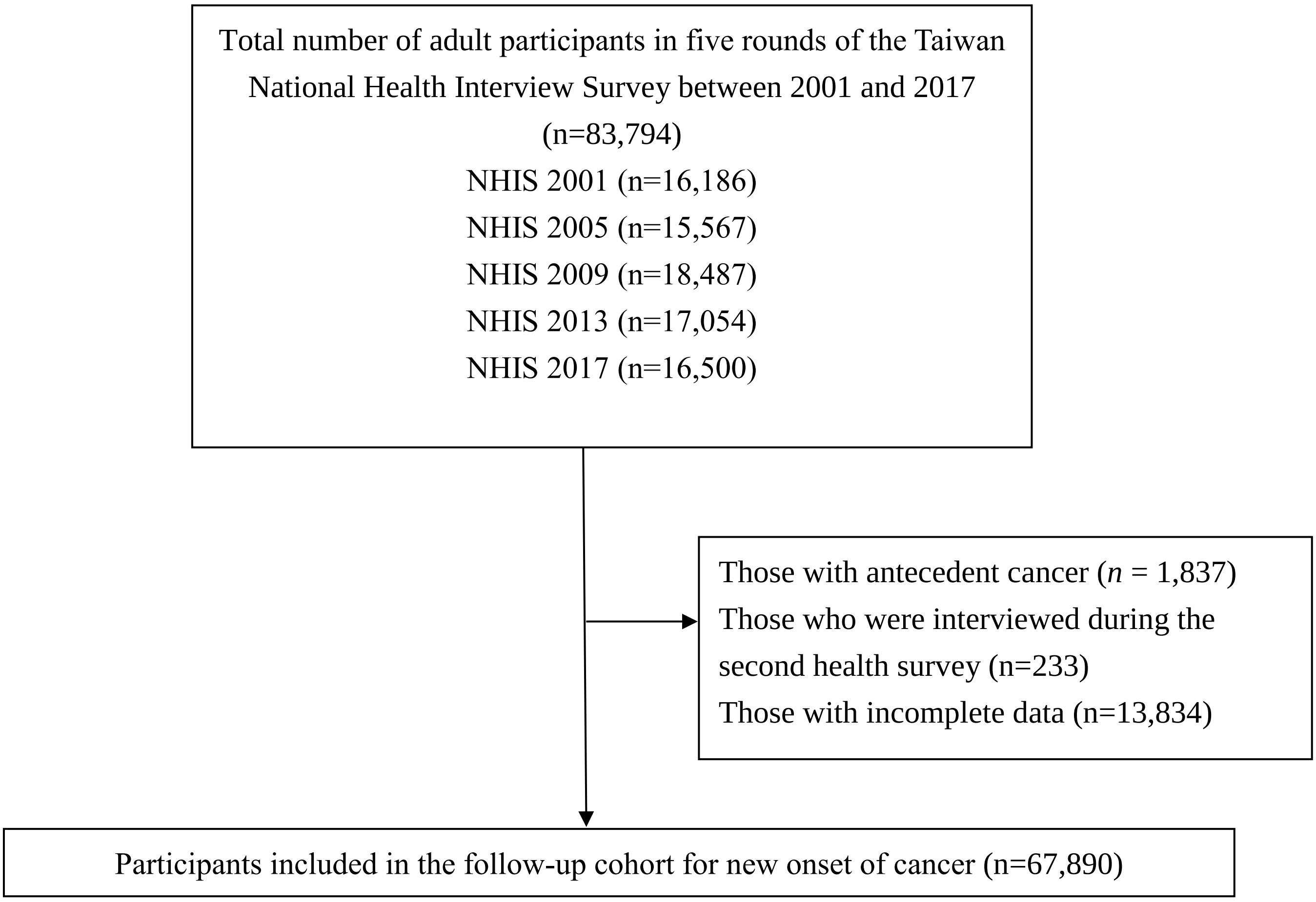
Total number of adult participants in five rounds of the Taiwan National Health Interview Survey between 2001 and 2017 (n=83,794)
NHIS 2001 (n=16,186)
NHIS 2005 (n=15,567)
NHIS 2009 (n=18,487)
NHIS 2013 (n=17,054)
NHIS 2017 (n=16,500)
Those with antecedent cancer (n = 1,837)
Those who were interviewed during the second health survey (n=233)
Those with incomplete data (n=13,834)
Participants included in the follow-up cohort for new onset of cancer (n=67,890)
